# The effect of algal polysaccharides laminarin and fucoidan on colonic pathology, cytokine gene expression and Enterobacteriaceae in a dextran sodium sulfate-challenged porcine model

**DOI:** 10.1017/jns.2016.4

**Published:** 2016-03-28

**Authors:** C. J. O'Shea, J. V. O'Doherty, J. J. Callanan, D. Doyle, K. Thornton, T. Sweeney

**Affiliations:** 1 School of Agriculture and Food Science, College of Life Sciences, University College Dublin, Belfield, Dublin 4, Republic of Ireland; 2 School of Veterinary Medicine, College of Life Sciences, University College Dublin, Belfield, Dublin 4, Republic of Ireland

**Keywords:** Laminarin, Fucoidan, Ulcerative colitis, Dextran sodium sulfate, Pigs, Cytokines, Enterobacteriaceae, DSS, dextran sodium sulphate, FUC, fucoidan, LAM, laminarin, LAMFUC, laminarin+ fucoidan, UC, ulcerative colitis

## Abstract

The algal polysaccharides laminarin (LAM) and fucoidan (FUC) have potent anti-inflammatory activities in the gastrointestinal tract. Our objective was to examine the impact of prior consumption of LAM and/or FUC on pathology and inflammation following a dextran sodium sulfate (DSS) challenge in pigs. Pigs (*n* 7/group) were assigned to one of five experimental groups for 56 d. From 49–55 d, distilled water or DSS was administered intragastrically. The experimental groups were: (1) basal diet + distilled water (control); (2) basal diet + DSS (DSS); (3) basal diet + FUC + DSS (FUC + DSS); (4) basal diet + LAM + DSS (LAM + DSS); and (5) basal diet + LAM + FUC + DSS (LAMFUC + DSS). The DSS group had decreased body-weight gain (*P* < 0·05) and serum xylose (*P* < 0·05), and increased proximal colon pathology score (*P* < 0·05), diarrhoeal score (*P* < 0·001) and colonic Enterobacteriaceae (*P* < 0·05) relative to the control group. The FUC + DSS (*P* < 0·01), LAM + DSS (*P* < 0·05) and LAMFUC + DSS (*P* < 0·05) groups had improved diarrhoeal score, and the LAMFUC + DSS (*P* < 0·05) group had improved body weight relative to the DSS group. The FUC + DSS group (*P* < 0·001), LAM + DSS group (*P* < 0·05) and LAMFUC + DSS group (*P* < 0·001) had lower *IL-6* mRNA abundance relative to the DSS group. The LAM + DSS group had reduced Enterobacteriaceae in proximal colon digesta relative to the DSS group (*P* < 0·05). In conclusion, FUC or a combination of FUC and LAM improved body-weight loss, diarrhoeal scores and clinical variables associated with a DSS challenge in pigs, in tandem with a reduction in colonic *IL-6* mRNA abundance.

Ulcerative colitis (UC) is a form of inflammatory bowel disease which has increased in incidence in recent years^(^^1^^,^^2^^)^. Clinically, UC is associated with bloody diarrhoea, abdominal cramps, weight loss and fatigue^(^^3^^)^. The inflammation pattern typical of UC is confined to the large intestine and is characterised histologically by alterations in the mucosal layer, including compromised epithelial integrity, goblet cell loss and infiltrations of immune cells^(^^3^^,^^4^^)^. The aetiology of UC is thought to reflect a failure of the host to achieve immune homeostasis in response to the presence of microbes in the colonic lumen. Features of this immune dysregulation may include excessive production of signalling pro-inflammatory cytokines, which can serve to initiate and perpetuate the chronic inflammatory pattern of UC. This aberrant immune response to the colonic microbiota may be exacerbated by the competitive advantage employed by enteric Enterobacteriaceae which can flourish under such conditions^(^^5^^)^ and may contribute to prolonged inflammation. Recent studies complicate this relationship further, indicating a possible earlier role for Enterobacteriaceae in inducing spontaneous colitis^(^^6^^)^. Therefore, the evidence for environmental and particularly microbial involvement in the genesis, severity and persistence of UC demonstrates the complexity of the disease.

The location of UC in the large intestine complicates the application of potential oral preventative and/or therapeutic solutions, which must first navigate the digestive processes of the upper gastrointestinal tract in order to influence the lumen environment. The algal-derived fibres laminarin (LAM), a β(1–3, 1–6)-linked glucan, and fucoidan (FUC), a sulfated fucose polymer, have shown immunomodulatory activity^(^^7^^)^, and improve clinical signs when included in the diet of abruptly weaned piglets^(^^8^^)^, which often undergo a period of gastrointestinal tract inflammation during dietary transition. The bioactivity of dietary LAM and FUC reflect in part the absence of appropriate endogenous host enzymes to hydrolyse these complex polysaccharides, which therefore arrive in the large intestine relatively intact and available for interaction with the lumen environment^(^^9^^,^^10^^)^. LAM and FUC demonstrate pleiotropic effects in the porcine large intestine including regulation of gene expression of pro- and anti-inflammatory cytokines relevant to UC, and inhibitory effects on the abundance of colonic Enterobacteriaceae^(^^8^^,^^11^^,^^12^^)^. These biological effects have been accompanied by improvements in nutrient absorption, body-weight gain and incidence of diarrhoea in piglets^(^^13^^,^^14^^)^. Thus, it was postulated that dietary LAM and/or FUC may alleviate the inflammation and underlying biological events which characterise UC. Oral exposure to dextran sodium sulfate (DSS) can induce many signs of UC^(^^15^^)^. The objective of the study was to investigate the preventative role of prior consumption of either LAM and FUC, or a combination of both, on the colonic pathology, inflammatory gene expression and abundance of Enterobacteriaceae following a DSS challenge in pigs.

## Materials and methods

All experimental procedures described in this study were conducted under experimental license from the Irish Department of Health in accordance with the cruelty to Animals Act 1876 and the European Communities (Amendments of the Cruelty to Animals Act, 1876) Regulations (1994).

### Experimental design and dietary composition

The study was executed as a complete randomised design comprising five experimental groups. The schedule consisted of a dietary exposure period commencing on the day of weaning (day 0) until euthanasia on day 56. From day 49 to day 55, pigs were administered either autoclaved water or a DSS solution (0·75 g/kg body weight; molecular weight 47·9 kDa; TdB Consultancy AB) via a single, daily oral administration. The experimental groups were: (1) basal diet + distilled water (control); (2) basal diet + DSS (DSS); (3) FUC diet + DSS (FUC + DSS); (4) LAM diet + DSS (LAM + DSS); and (5) LAM + FUC diet + DSS (LAMFUC + DSS). The concentrations of dietary LAM (300 mg/kg) and FUC (240 mg/kg) used were based on previous optimisation studies^(^^16^^,^^17^^)^. The LAM (990 g LAM/kg) and FUC (720 g FUC/kg, 180 g/kg crude protein and 100 g/kg ash) were derived from *Laminaria* spp. (BioAtlantis Ltd). The diets were offered *ad libitum* from day 0 until day 56, after which time pigs were euthanised to facilitate gastrointestinal tract tissue and digesta recovery. The diets were formulated to provide digestible energy (14·5 MJ/kg) and standardised ileal digestible lysine content (12·5 g/kg) appropriate to the age and growth rate of the pigs^(^^18^^)^. The ingredient composition and chemical analysis of the dietary treatments are presented in Supplementary Table S1.

### Animals and management

A total of thirty-five pigs (progeny of Large White × (Large White × Landrace) sows) weighing 7 (sd 0·53) kg were assigned to one of five experimental groups (*n* 7 pigs/experimental group) and were individually housed in pens (1·7 × 1·2 m). Feed and water were provided *ad libitum*. The pigs were weighed at the beginning of the experiment (day 0), at the commencement of DSS dosing (day 49) and the day of euthanasia (day 56) to obtain body weight and calculate average daily body-weight change.

### Diarrhoeal score

All pigs were individually observed for clinical signs of diarrhoea from day 49 to day 56 of the experiment by a single operator with no prior knowledge of experimental groups. A scoring system was applied to indicate presence and extent of severity^(^^19^^)^. The following scoring system was used: 1 = hard firm faeces; 2 = slightly soft faeces; 3 = soft, partially formed faeces; 4 = loose, semi-liquid faeces (diarrhoea); and 5 = watery, mucous-like faeces (severe diarrhoea).

### Feed and seaweed extract compositional analysis

Representative feed samples were collected at regular intervals throughout the experiment. The DM of feed samples was determined after drying at 103°C for 16 h. For compositional analysis, feed samples were milled through a 1 mm screen (Christy and Norris Hammer Mill). The crude ash content of the feed samples was determined by combustion using a muffle furnace (Nabertherm) at 500°C for 4 h. The N content of the feed was determined by combustion using a LECO FP 528 instrument (Leco Instruments Ltd) and was used to estimate dietary crude protein content (N × 6·25). The neutral-detergent fibre fraction of feed samples was determined using a fibretec extraction unit (Tecator), while the gross energy content was determined by bomb calorimetry using a Parr 1201 oxygen bomb calorimeter (Parr Instrument Co.). The LAM content of the supplements and the feed samples was determined by spectrophotometry using a commercial assay kit (Megazyme Ireland Ltd) as previously described^(^^20^^)^. The FUC content of the supplements and feed samples was determined using the method of Usov *et al*.^(^^21^^)^. Briefly, this procedure involved acid hydrolysis (0·2 m-HCl) of the ground algal biomass, decolourisation of the acid extract using sodium chlorite, and spectrophotometric determination of FUC content after the removal of inorganic salts by dialysis.

### Serum xylose, small intestinal and colonic histopathological analysis

**Fig. 1.**
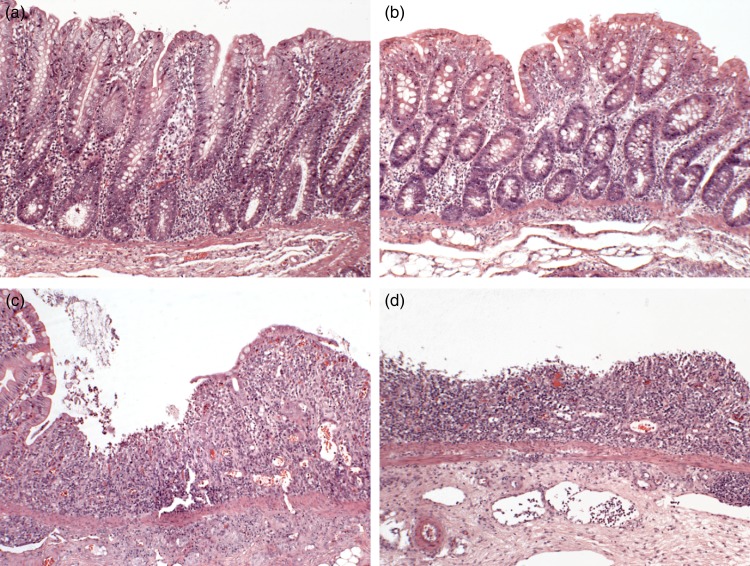
Representative sections of proximal colon stained with haematoxylin and eosin (magnification × 10) demonstrating the spectrums of pathology. (a) Healthy tissue, (b) healthy with a reduced lamina proprial cell infiltrate, (c), focal ulceration bordered by intact epithelium, (d) diffuse ulceration. The histology shown is representative of the colon tissues studied in all five groups of piglets (*n* 7 per group).

On day 56, pigs were administered a single dose of xylose (d-(+)-xylose; 0·5 g/kg body weight; Sigma-Aldrich) 2 h prior to euthanasia by oral administration to determine subsequent serum xylose as a marker of small-intestinal absorption^(^^22^^)^. Serum was collected at euthanasia and serum xylose was assessed by spectrophotometry using a commercial assay kit (Megazyme). Pigs were humanely euthanised by lethal injection with pentobarbital sodium (Euthatal, Merial Animal Ltd) at a rate of 1 ml/1·4 kg body weight. Immediately after slaughter, the entire digestive tract was removed. Consistent sections of the ileum (10 cm from the ileo-caecal valve), the proximal colon (2nd loop from the caeco-colic junction) and the distal colon (3rd loop from the rectum) were excised and fixed in 10 % neutral buffered formalin. Following fixation, tissues were trimmed and paraffin-embedded. The samples were then sectioned at a 5 µm thickness and stained with haematoxylin and eosin. For the ileum, villous height and crypt depth were measured on the stained sections using a light microscope fitted with an image analyser (Image-Pro Plus; Media Cybernetics). Measurements of fifteen well-oriented villi and crypts were taken for each segment. Villous height was measured from the crypt–villous junction to the villous tip. Crypt depth was measured from the crypt–villous junction to the crypt base. Results are expressed as mean villous height or crypt depth in μm. For histopathological assessment, the proximal and distal colon histology sections were examined in blinded fashion by a board-certified pathologist (J. J. C.) to evaluate the extent of ulceration as defined by loss of colonic epithelial cells and an associated variation in the concentrations of lamina proprial cells^(^^23^^,^^24^^)^. A histological scoring scheme was used to qualify the extent of colonic ulceration damage ranging from histological features consistent with a normal colon, occasionally with evidence of reduced lamina proprial cell infiltrate, to a severe UC characterised by diffuse epithelial cell loss, crypt obliteration and lamina proprial inflammatory cell infiltration which were usually mononuclear with occasional neutrophils and eosinophils. The scoring system primarily focused on the extent of ulceration, as it was beyond the scope of this study to phenotype the lymphoid, histiocytic, plasma cell lamina proprial infiltrations. Score 1 was considered normal, non-ulcerating epithelium with a reduced lamina proprial cell infiltration in comparison with score 2 which was normal with a lamina proprial cell infiltration within the normal spectrum. Score 3 represented colonic samples with focal (single) regions of ulceration and scores 4 and 5 represented colonic samples with multiple foci ulceration interspersed between regions of normal colon and samples with diffuse ulceration respectively. These latter colons had obliteration of crypt architecture and evidence of acute inflammation with prominent neutrophil margination within blood vessels in the lamina propria. Examples of representative samples assigned to various pathology scores are presented in Fig. 1.

### Proximal colon gene expression

Tissue in the proximal colon was excised, rinsed with sterile PBS (Oxoid), stripped of smooth muscle, and stored in RNAlater. Total RNA was isolated in Tri reagent (Sigma-Aldrich) using a phenol–chloroform method and column-purified (GenElute Mammalian Total RNA Miniprep kit; Sigma-Aldrich) incorporating an on-column residual DNA digestion step (Deoxyribonuclease I; Sigma-Aldrich). Quality and quantity of RNA was assessed using a NanoDrop (Thermo Fisher Scientific) and an Agilent 2100 Bioanalyzer with an RNA 6000 Nano LabChip kit (Agilent Technologies). All samples had a 260:280 ratio greater than 2·0 and RNA integrity numbers (RIN) greater than 8·0. Synthesis of a cDNA library was performed on 1 µg of total RNA using a high-capacity cDNA reverse transcription kit (Applied Biosystems). Primers targeting selected cytokine and transcription factors (Supplementary Table S2) were designed using Primer Express™ (PE Applied Biosystems) and synthesised by MWG Biotech. The reference genes β−2-microglobulin (*B2M*), glyceraldehyde-3-phosphate dehydrogenase (*GAPDH*), β-actin (*ACTB*), hydroxymethylbilane synthase (*HMBS*), peptidylprolyl isomerase (*PPIA*) and tyrosine 3-monooxygenase/tryptophan 5-monooxygenase activation protein *ξ* polypeptide (*YWHAZ*) were evaluated for stability using geNORM^(^^25^^)^. The reference genes *PPIA* and *YWHAZ* were selected as the most stably expressed in colonic tissue, and were used for subsequent normalisation. Amplification was carried out in a reaction volume of 20 µl containing 10 µl of SYBR Green MasterMix (Applied Biosystems), 300 nm of each forward and reverse primer, 8 µl of nuclease-free water and 1 µl of template cDNA. Quantitative PCR was carried out using an ABI PRISM 7500 Fast Sequence Detection System (Applied Biosystems). Thermal cycling conditions were as follows: an initial denaturation step at 95°C for 20 s, forty cycles of denaturation at 95°C for 15 s followed by 60°C for 1 min. All samples were run in triplicate and dissociation analysis of the PCR products was performed to confirm specificity. Relative quantities for each target gene were normalised using the normalisation factor calculated by the qbase^+^ algorithm (Biogazelle NV). The normalisation factor is the geometric mean of the most stable endogenous controls^(^^26^^)^.

### Microbiology

Microbial genomic DNA was extracted from proximal colon digesta using a QIAamp DNA stool kit (Qiagen) in accordance with the manufacturer's instructions. Quantity and quality of DNA were assessed using a Nanodrop (Thermo Scientific). Standard curves were prepared from pooled aliquots of digesta microbial DNA and used to estimate absolute numbers of colonic Enterobacteriaceae based on gene copy number^(^^27^^)^. For the quantitative real-time PCR assay, oligonucleotide primers specific to the Enterobacteriaceae 16 s rRNA gene (forward primer 5′−3′ CATTGACGTTACCCGCAGAAGAAGC and reverse primer 5′−3′ CTCTACGAGACTCAAGCTTGC) were used^(^^28^^)^. Real-time PCR using an ABI 7500 Real-Time PCR System (Applied Biosystems) was performed in a final reaction volume of 20 µl containing 1 µl template DNA, 1 µl of forward and reverse primers (100 pm), 10 µl SYBR Green PCR Master Mix (Applied Biosystems) and 8 µl nuclease-free water. The thermal cycling conditions involved an initial denaturation step at 95°C for 10 min followed by forty cycles of 95°C for 15 s and 65°C for 1 min. Dissociation curve analyses of the PCR product confirmed the specificity of the assay. The mean threshold cycle values from triplicates of each sample were used for calculations.

### Statistical analysis

The body weight, serum xylose, diarrhoea score, cytokine, Enterobacteriaceae and SCFA data were analysed as a complete randomised design using the general linear model procedure of SAS^(^^29^^)^. Least square means between experimental groups were compared using preplanned contrast statements. Preplanned comparisons between experimental groups were as follows: 1, DSS *v*. control; 2, FUC + DSS *v*. DSS group; 3, LAM + DSS *v*. DSS group; and 4, LAMFUC + DSS *v*. DSS group. Data were checked for normality using the univariate procedure of SAS^(^^29^^)^.The body-weight data were adjusted for weaning weight by covariance analysis. Estimations of gene copy numbers of Enterobacteriaceae were log-transformed before statistical analysis and are presented as gene copy numbers per g digesta. Differences in histopathological score data between experimental groups were analysed using Fisher's exact test with the frequency procedure of SAS^(^^29^^)^. Pearson and Spearman correlation coefficients amongst colon pathology score, body-weight variables and cytokine mRNA abundance were determined using the correlation procedure of SAS^(^^29^^)^. The probability level that denotes significance is *P* < 0·05. The data in the tables are presented as least square means and standard errors.

## Results

### Body-weight variables, diarrhoea scoring and serum xylose

Initial body weight, body-weight gain during the DSS experimental period (kg gain/day 49–day 56), and final body weight (day 56) are presented in Table 1. There was no significant difference between experimental groups on body weight of pigs before commencement of dosing with DSS (day 49). The DSS group had a lower body-weight gain (*P* < 0·05) and a lower final body weight on day 56 (*P* < 0·05) compared with the control group. The LAMFUC + DSS group had a higher final body weight on day 56 (*P* < 0·05) when compared with the DSS group.

**Table 1.** Effect of prior dietary exposure to laminarin (LAM) and/or fucoidan (FUC) and subsequent dosing with water or dextran sodium sulfate (DSS) on body weight, serum xylose, diarrhoeal score and ileal morphology of pigs (Least squared means (*n* 7/experimental group) with their standard errors)

	Treatment						Contrasts†			
	Control	DSS	FUC + DSS	LAM + DSS	LAMFUC + DSS	sem	1	2	3	4
Performance										
Initial body weight (kg)	18·5	17·6	18·3	18·5	18·8	0·49	NS	NS	NS	NS
Body-weight gain (kg/d)‡	0·831	0·581	0·628	0·419	0·660	0·07	*	NS	NS	NS
Final body weight (kg)§	24·3	21·7	22·7	21·4	23·4	0·56	*	NS	NS	*
Diarrhoeal score	2·5	4·7	3·5	4·0	4·2	0·16	***	**	*	*
Gut absorption										
Serum xylose (mg/l)||	57·8	28·6	43·0	38·5	40·6	8·3	*	NS	NS	NS
Ileal morphology										
Villous height (μm)	325·5	330·2	318·3	311·2	300·8	19·6	NS	NS	NS	NS
Crypt depth, (μm)	307·0	288·6	278·4	311·3	294·7	21·4	NS	NS	NS	NS
Villus height:crypt depth	1·1	1·1	1·1	1·0	1·0	0·09	NS	NS	NS	NS

LAMFUC, laminarin + fucoidan; NS, *P* ≥ 0·05.

* *P* < 0·05, ** *P* < 0·01, *** *P* < 0·001.

† Preplanned contrasts with *P* value for comparison: contrast 1 = DSS *v*. control; contrast 2 = FUC + DSS *v*. DSS; contrast 3 = LAM + DSS *v*. DSS; contrast 4 = LAMFUC + DSS *v*. DSS.

‡ Average body-weight gain of pigs (kg/d) from commencement to conclusion of dosing with DSS (day 49–day 56).

§ Final body weight of pigs upon conclusion of dosing with DSS (day 56).

|| Serum xylose on day of euthanasia (day 56).

From day 52 to day 56 of DSS administration, the DSS group had a higher average diarrhoeal score compared with the control group (*P* < 0·001). From day 52 to day 56 of DSS administration, the DSS group also had a higher average diarrhoeal score compared with the FUC + DSS group (*P* < 0·01), the LAM + DSS group (*P* < 0·05) and the LAMFUC + DSS group (*P* < 0·05).

The DSS group had a lower serum xylose when compared with the control group (*P* < 0·05). The DSS group tended to have a lower serum xylose (*P* < 0·1) when compared with the LAMFUC + DSS group.

### Ileal morphology and proximal and distal colon pathology

**Fig. 2.**
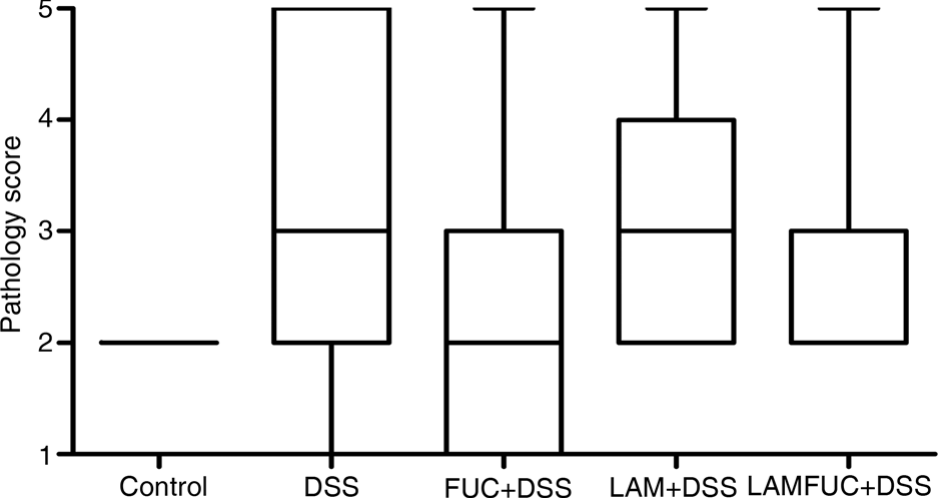
Pathology score in the proximal colon of experimental groups receiving experimental diets for 56 d and exposed to water or dextran sodium sulfate (DSS) from day 49 to day 55 (*n* 7/experimental group). Control, basal diet + distilled water, DSS, basal diet + DSS, FUC + DSS, fucoidan diet + DSS; LAM + DSS, laminarin diet + DSS; LAMFUC + DSS, laminarin + fucoidan diet + DSS. The scoring system was as follows: 1, normal with reduced lamina proprial cell infiltrate; 2, normal; 3, focal ulceration; 4, multi-focal ulceration; 5, diffuse ulceration. The box and whisker plots represent the medians, and first and third quartiles; the vertical bars are ranges.

In the ileum, there was no significant difference between experimental groups on villus height, crypt depth or the villus height:crypt depth ratio (Table 1). In both the proximal colon (Fig. 1) and distal colon, the control group was described as having histological features consistent with normality. A spectrum of pathology was observed in the proximal colon and distal colon (data not presented) of experimental groups receiving DSS which ranged from healthy through to diffuse ulceration. Additionally, a subset of the population was deemed to display healthy histology but with a reduced immune cell infiltration of the lamina propria. These animals were predominately within the FUC + DSS group. In the proximal colon, the DSS group had higher pathology scores when compared with the control group (Fig. 2; *P* < 0·05). The pathology scores of the proximal colon from pigs assigned to the FUC + DSS, LAM + DSS and LAMFUC + DSS groups were not significantly different from either the DSS group or the control group. Finally, in the distal colon, the pathology score was not significantly different between experimental groups (data not presented).

### Cytokine gene expression in the proximal colon

In the proximal colon, the DSS group had increased mRNA abundance of *IL-6* (*P* < 0·001) when compared with the control group (Table 2). The FUC + DSS group had decreased mRNA abundance of *IL-6* (*P* < 0·001) and a tendency for a decreased mRNA abundance of *IL-8* (*P* < 0·1) when compared with the DSS group. The LAM + DSS group had a decreased abundance of *IL-6* (*P* < 0·05) and a tendency for decreased mRNA abundance of *IL-8* (*P* < 0·1) when compared with the DSS group. The LAMFUC + DSS group had a decreased mRNA abundance of *IL-6* (*P* < 0·001) and *IL-8* (*P* < 0·05) when compared with the DSS group.

**Table 2.** Effect of prior dietary exposure to laminarin (LAM) and/or fucoidan (FUC) and subsequent dosing with water or dextran sodium sulfate (DSS) on normalised relative mRNA abundance of selected cytokines in proximal colon tissue (Least squared means (*n* 7/experimental group) with their standard errors)

	Treatment						Contrasts†			
	Control	DSS	FUC + DSS	LAM + DSS	LAMFUC + DSS	sem	1	2	3	4
*IL-6*	0·455	0·937	0·318	0·609	0·381	0·09	***	***	*	***
*IL-1β*	0·678	0·949	0·667	0·736	1·118	0·20	NS	NS	NS	NS
*IL-8*	0·750	0·915	0·448	0·454	0·390	0·16	NS	0·1	0·1	*
*IL-17A*	0·954	1·037	0·761	1·138	1·638	0·27	NS	NS	NS	NS
*IL-10*	0·950	1·112	0·481	0·482	1·616	0·31	NS	NS	NS	NS
*TGF-β*	1·07	0·900	0·496	0·784	1·307	0·18	NS	NS	NS	NS
*TNF-α*	0·702	0·844	0·523	0·552	1·777	0·40	NS	NS	NS	NS
Enterobacteriaceae (gene copies/g digesta)	8·19	9·23	8·58	8·21	8·50	0·31	*	NS	*	NS

LAMFUC, laminarin + fucoidan; NS, *P* ≥ 0·05.

* *P* < 0·05, *** *P* < 0·001.

† Preplanned contrasts with *P* value for comparison: contrast 1 = DSS *v*. control; contrast 2 = FUC + DSS *v*. DSS; contrast 3 = LAM + DSS *v*. DSS; contrast 4 = LAMFUC + DSS *v*. DSS.

### Enterobacteriaceae in the proximal colon digesta

In the proximal colon, the DSS group had increased 16 s rRNA gene copy numbers of Enterobacteriaceae (*P* < 0·05) when compared with the control group (Table 2). The LAM + DSS group had decreased Enterobacteriaceae (*P* < 0·05) when compared with the DSS group.

### Correlation analysis

Correlation coefficients between body-weight variables, colon pathology scores and proximal colon cytokine gene expression are presented in Table 3. Final body weight (day 56) was negatively associated with proximal colon pathology score (*r* −0·58; *P* < 0·001) and distal colon pathology score (*r* −0·43; *P* < 0·05). Body-weight gain during administration of DSS (day 49–day 56) was negatively associated with proximal colon pathology score (*r* −0·75; *P* < 0·001) and distal colon pathology score (*r* −0·74; *P* < 0·001). Final body weight was negatively correlated with gene expression of *IL-6* (*r* −0·48; *P* < 0·05), *IL-1β* (*r* −0·52; *P* < 0·01), *IL-17A* (*r* −0·39; *P* < 0·05) and *TNF-α* (*r* −0·43; *P* < 0·05). Body-weight gain was negatively associated with gene expression of *IL-6* (*r* −0·62; *P* < 0·001) and *IL-1β* (*r* −0·49; *P* < 0·05). Proximal colon pathology score was positively associated with gene expression of *IL-6* (*r* 0·86; *P* < 0·001) and positively associated with gene expression of *IL-8* (*r* 0·41; *P* < 0·05), *IL-1β* (*r* 0·58; *P* < 0·001), *IL-17A* (*r* 0·38, *P* < 0·05), *TGF-β* (*r* 0·48; *P* < 0·01), *TNF-α* (*r* 0·56; *P* < 0·01) and *IL-10* (*r* 0·52; *P* < 0·01). Finally, proximal colon and distal colon pathology scores were positively correlated (*r* 0·77; *P* < 0·001).

**Table 3.** Pearson and Spearman correlation coefficients for associations between body weight, colonic pathology score and relative mRNA abundance of cytokines in the proximal colon of pigs†‡

	Proximal colon gene expression of selected inflammatory markers							Pathology score	
	*IL-8*	*IL-6*	*IL-1β*	*IL-17A*	*TGF-β*	*TNF-α*	*IL-10*	Proximal colon	Distal colon
Final body weight	−0·18	−0·48*	−0·52**	−0·39*	−0·19	−0·43*	−0·15	−0·58***	−0·43*
Body-weight gain	−0·23	−0·62***	−0·49*	−0·34	−0·27	−0·29	−0·15	−0·75***	−0·74***
Pathology score (proximal colon)	0·41*	0·86***	0·58***	0·38*	0·48**	0·56**	0·52**		0·77***

Significant correlation coefficients: * *P* < 0·05, ** *P* < 0·01, *** *P* < 0·001.

† Spearman's rank correlation coefficients were used for determination of statistical dependence between pathology score and other variables, and Pearson's correlations for relationships between body weight and cytokine data.

‡ For correlation analysis graphs, see Supplementary Fig. S1.

## Discussion

In the present study, under a DSS challenge situation, pigs had impaired body-weight gain and diarrhoea. This was accompanied by histopathological evidence of UC in the proximal colon, increases in pro-inflammatory cytokine gene expression, and an increase in the luminal abundance of Enterobacteriaceae. Previously, the algal polysaccharides LAM and FUC have been shown to exhibit anti-inflammatory activity, and also inhibitory effects on Enterobacteriaceae^(^^11^^,^^12^^,^^30^^)^, and were investigated here for a potential role in alleviating the effects of a DSS challenge period in pigs. Prior consumption of FUC and/or LAM improved the diarrhoeal score associated with a DSS challenge, indicating a protective effect. These effects were accompanied by a decrease in the gene expression of the proinflammatory cytokine *IL-6* mRNA abundance in proximal colonic tissue. Additionally, prior consumption of LAM alone reduced the abundance of Enterobacteriaceae in the proximal colon associated with a DSS challenge.

The development of UC is frequently associated with weight loss, due to a combination of inappetence, malabsorption of nutrients, and the loss of fluid and electrolytes through diarrhoea^(^^3^^,^^31^^)^. The final body weight of the DSS group was approximately 11 % less than that of the control group at the end of the experiment, reflecting a reduction in body-weight gain of 30 % during the 7 d DSS administration period. Previous studies have shown that dietary inclusion of seaweed extract containing LAM and FUC can improve body-weight gain in newly weaned piglets, which often undergo transient colonic inflammation due to the stress of dietary changes^(^^13^^,^^14^^)^. The present results show that the LAMFUC + DSS group had a greater final body weight at the end of the experiment relative to the DSS group, indicating an alleviation of the weight loss associated with a DSS challenge. However, while the FUC + DSS group had improved weight gain, neither that group nor the LAM + DSS group was different from the DSS group, suggesting that the seaweed polysaccharide extracts are more effective in combination in preserving body weight.

While diarrhoea is an important component of the innate immune system, facilitating pathogen clearance from colonic epithelium^(^^32^^,^^33^^)^, it is of limited value in UC, serving to weaken the host through depleted fluids and electrolytes. In this study, the DSS group developed diarrhoea following the administration of DSS for 7 d. Interestingly, the experimental groups containing LAM and FUC both singly and collectively reduced the severity of diarrhoea associated with a DSS challenge. Of these, the FUC + DSS group displayed the greatest amelioration of diarrhoea relative to the DSS group. In an earlier study, Zhang *et al*.^(^^34^^)^ showed that intravenous administration of FUC ameliorated mucosal damage in a murine model of colitis. The roles of these seaweed extracts, and particularly FUC, in alleviating diarrhoea merits further investigation given the contribution that persistent diarrhoea has to the health burden of patients with UC^(^^35^^)^.

In UC, pathology typically occurs between the ileocaecal valve and the rectum. However, there may be some involvement with the distal ileum, and such ileitis results in mucosal erosion and stunted villi^(^^36^^)^, contributing to malabsorption of nutrients, and weight loss^(^^3^^,^^37^^)^. In this study, there was no evidence of villus dystrophy observed in the ileum of the DSS group relative to the control group; however, the serum xylose concentrations of the former were lower when compared with the latter, suggesting some degree of small-intestine xylose malabsorption^(^^38^^)^. Therefore, although DSS-induced inflammation was localised to the large intestine, the function of the ileum may have been compromised due to moderate aggravation by DSS^(^^39^^)^. Recently we showed that seaweed extracts rich in LAM and FUC improve the digestibility of nutrients in pigs undergoing a weaning stress^(^^13^^)^, possibly due to up-regulation of genes involved in monosaccharide transport^(^^40^^)^. However, in the present study, the serum xylose concentrations of all DSS groups receiving seaweed extracts were higher, but not different between the control and DSS groups.

Microscopic examination of the proximal and distal colon identified a spectrum of pathology in the DSS group at both sites indicating widespread onset of colonic ulceration and inflammation. In the proximal colon, the DSS group had a greater pathology score relative to the control group, indicating this region to be the main site of DSS-induced inflammation. In the distal colon, the pathology score was higher, but not different in the DSS group relative to the control group, hence further comparative analysis between experimental groups focused on the proximal colon. In the proximal colon, the DSS group exhibited a spectrum of pathology which was characterised by single, multi-focal or diffuse regions of colonic ulceration. Accordingly, the aforementioned body-weight gain during the DSS period was negatively associated with the pathology score of both the proximal (*r* −0·75) and distal colon (*r* −0·74), with a lower body-weight gain commensurate with a more severe colonic pathology score. The pathology scores in the proximal colon of experimental groups receiving LAM and/or FUC tended to be intermediate, but not different between the control group and the DSS group. Qualitative histopathological differences indicating reduced immune cell infiltrate were noted in only a subset of animals in the FUC + DSS group. These findings are contrary to the observations of Zhang *et al*.^(^^34^^)^ who obtained stronger evidence of a protective role for FUC in protecting against DSS-induced colitis in a murine model.

The incidence of inflamed tissue as observed in active UC is typically accompanied by an elevated pattern of pro-inflammatory cytokines, which serve to initiate and perpetuate inflammatory processes in the colonic mucosa^(^^41^^)^. In the present study, the mRNA abundances of a select panel of pro- and anti-inflammatory cytokines were all positively correlated with pathology score, in agreement with the observations of previous studies investigating the contribution of inflammatory cytokines in the DSS model of UC^(^^42^^,^^43^^)^. The observation of a moderate and positive correlation between mRNA abundance of pro-inflammatory cytokines such as *IL-17A, IL-8* and *TNF-α* and proximal colon pathology score is an expected association, given the role such inflammatory mediators play in stimulating and amplifying the pattern of chronic inflammation characteristics of DSS models of UC^(^^43^^)^. Furthermore, the positive association of anti-inflammatory cytokines such as *IL-10*, and *TGF-β* which can act in an anti-inflammatory capacity, with pathology score may also be expected to be elevated in inflamed tissue, acting to quell inflammatory processes. An increase in these cytokines has previously been observed in comparable models of UC^(^^43^^–^^45^^)^. The most pronounced positive association observed for mRNA abundance of a cytokine with proximal colon pathology score was for *IL-6* (*r* 0·86), a pro-inflammatory cytokine which has been implicated in playing a considerable role in UC, and previously shown to strongly and positively correlate with the severity of disease observed in UC using the Mayo Scoring System^(^^46^^)^. Reflecting this, the DSS experimental group had increased mRNA abundance of *IL-6* in the proximal colon relative to the control group. The FUC + DSS and LAMFUC + DSS groups had a 2·9- and 2·5-fold lower mRNA abundance of *IL-6* respectively, relative to the DSS group. In other studies, Bahar *et al*.^(^^47^^)^ showed that FUC-rich algae extracts can inhibit the mRNA abundance of *IL-6* induced by lipopolysaccharide in *ex vivo* porcine tissue. These observations reflect findings from another study by Matsumoto *et al*.^(^^48^^)^ who showed a comparable effect of algal-derived FUC in regulating *IL-6* in colonic epithelial cells and which in turn alleviated colitis in a murine model. The LAMFUC + DSS group also had a reduced mRNA abundance of *IL-8* relative to the DSS group; however, this was not observed when FUC was offered alone, consolidating a pattern in the present study of diverse activity of FUC and LAM in the absence or presence of the other. The LAM + DSS group had decreased mRNA abundance of *IL-6* and a tendency for decreased mRNA abundance of *IL-8* relative to the DSS group and overall had a lesser effect on cytokine gene expression when offered singly rather than when combined with FUC.

The role of the enteric microbiota has drawn much attention due to inherent differences in various bacterial taxonomic strata in the profile of UC patients when compared with that of healthy test subjects^(^^49^^)^. For example, there is more evidence for increased abundance of Gram-negative bacteria in patients presenting with UC in some studies^(^^50^^)^ including Enterobacteriaceae, which have been associated with the severity of disease^(^^51^^)^. In the present study, the DSS group had a 1 log-fold increase in Enterobacteriaceae in the proximal colon relative to the control group, paralleling the association of Enterobacteriaceae with UC observed in clinical research. It has previously been shown that LAM and FUC can modify the profile of the enteric microbiota, including reducing the abundance of Enterobacteriaceae in porcine colonic digesta^(^^8^^,^^20^^)^. The LAM + DSS group had a decreased abundance of Enterobacteriaceae relative to the DSS group, which was at a level comparable with the control group. The significance of this effect within the context of how LAM mitigated other measured variables associated with a DSS challenge in the present study is not clear, but may be important given the concern of a role for Enterobacteriaceae in inflammatory bowel disease.

In summary, a DSS challenge in the pig was associated with decreased body weight, diarrhoea and increases in the pro-inflammatory cytokine *IL-6*, and also in the abundance of Enterobacteriaceae in colonic digesta. The findings of this study show that prior exposure to diets containing FUC and a combination of FUC and LAM together, ameliorated weight loss, diarrhoea, but failed to improve the pathology score associated with a DSS challenge in the proximal colon of pigs. A strong correlation between mucosal mRNA abundance of *IL-6* with the severity of pathology in the proximal colon was observed. Interestingly, experimental groups receiving both LAM and/or FUC prior to the onset of a DSS challenge had decreased *IL-6* mRNA abundance, which may underpin the protective effect bestowed by these algal polysaccharides against a DSS challenge. Furthermore, the LAM + DSS group had a reduced abundance of Enterobacteriaceae associated with a DSS challenge. Collectively, the data suggest that prior consumption of LAM and FUC provide some evidence of a possible role for these algal polysaccharides in nutritional therapy for UC. However, more research is required to establish the capacity for effective protection against the development of pathology associated with UC.

## Supplementary material

The supplementary material for this article can be found at http://www.journals.cambridge.org/10.1017/jns.2016.4
